# Shared neural signatures of photophobia in migraine and post-traumatic headache: a task-based fMRI study

**DOI:** 10.1186/s10194-025-02088-y

**Published:** 2025-07-03

**Authors:** Rune Häckert Christensen, Haidar Muhsen Al-Khazali, Anna Gudrun Melchior, Messoud Ashina, Håkan Ashina

**Affiliations:** 1 Department of Neurology, Danish Headache Center, Copenhagen University Hospital – Rigshospitalet, Copenhagen, Denmark; 2 Department of Clinical Medicine, Faculty of Health and Medical Sciences, University of Copenhagen, Copenhagen, Denmark; 3 Translational Research Center, Copenhagen University Hospital – Rigshospitalet, Copenhagen, Denmark

**Keywords:** Post-Traumatic headache, Migraine, Headache, Functional magnetic resonance imaging, Sensory processing

## Abstract

**Background:**

Persistent post-traumatic headache (PTH) and migraine frequently present with photic hypersensitivity that exacerbates headache symptoms. We sought to determine whether persistent PTH is associated with altered brain responses to visual stimuli and to explore shared neural mechanisms of photophobia with migraine.

**Methods:**

This cross-sectional functional magnetic resonance imaging (fMRI) study included 80 adults with persistent PTH, 261 with migraine, and 143 healthy controls (HCs). All participants underwent visual stimulation using a flickering checkerboard during a 3T fMRI session. Blood oxygen level-dependent (BOLD) responses were examined using whole-brain and region-of-interest (ROI) analyses. All analyses were adjusted for age and sex.

**Results:**

Whole-brain analysis revealed no significant BOLD differences across the full persistent PTH, migraine, and HC groups. However, participants with persistent PTH who experienced photophobia during the scan (*n* = 41) showed greater activation in the anterior and midcingulate cortex compared with HCs (*P*_FWE_ = 0.010). No differences were observed between photophobic participants with persistent PTH and those with migraine who reported an attack during the fMRI session. ROI analyses identified greater activation in the anterior cingulate, midcingulate, and insular cortices in both photophobic participants with persistent PTH and ictal participants with migraine, relative to HCs (all *P* < 0.05). No significant differences were found between photophobic participants with persistent PTH and ictal participants with migraine.

**Conclusions:**

Photophobia in persistent PTH is associated with greater activation in cortical regions implicated in pain processing. These patterns parallel those observed during migraine attacks, indicating shared neural mechanisms between the two headache disorders.

**Supplementary Information:**

The online version contains supplementary material available at 10.1186/s10194-025-02088-y.

## Introduction

Persistent post-traumatic headache (PTH) is a prevalent and often disabling sequela of mild traumatic brain injury (mTBI), affecting many patients beyond 12 months post-injury [1, 2]. Clinically, persistent PTH closely resembles migraine, with shared features such as recurrent moderate-to-severe headache, photophobia, phonophobia, and nausea [3–5]. These striking similarities raise important questions about potential shared pathophysiological mechanisms [1, 6].

One such potential mechanism is altered sensory processing, a well-established feature of migraine that might also be relevant in persistent PTH [7]. In migraine, this alteration is thought to reflect hyperexcitability in specific brain regions [8, 9]. Our recent functional magnetic resonance imaging (fMRI) study found that visual stimulation during migraine attacks caused greater activation in the anterior cingulate and insular cortices, compared with healthy controls (HCs) [10]. These areas are implicated in both sensory integration and pain processing [11–14], which might explain the discomfort caused by light, often reported during migraine attacks [10]. Notably, increased visual cortex activation was observed in people with migraine even when headache-free, suggesting persistent sensory changes.

Despite these insights, it remains unclear whether similar alterations in sensory processing occur in persistent PTH. This uncertainty stems in part from the limited number of neuroimaging studies examining functional brain changes in persistent PTH [15–17]. A recent 2025 systematic review highlighted the absence of task-based fMRI studies in this population [18].

To address this gap, we conducted a cross-sectional task-based fMRI study involving adults with persistent PTH, migraine, and HCs. A standardized visual stimulation paradigm was used to assess sensory processing. Blood oxygen level-dependent (BOLD) responses were compared using both whole-brain and region-of-interest (ROI) analyses. We hypothesized that participants with persistent PTH would show altered BOLD activity relative to controls, particularly in regions related to pain and sensory integration. We also expected these responses to resemble those observed in participants with migraine.

## Methods

The study protocol was approved by the Ethics Committee of the Capital Region of Denmark (H-20033264), and written informed consent was obtained from all participants prior to any study-related procedures. The study was conducted in accordance with the Declaration of Helsinki. The full MRI protocol is available on ClinicalTrials.gov (Identifier: NCT04674020).

Participants were enrolled and scanned between November 2020 and October 2023. The study included adults with persistent PTH. For comparison, we included participants with migraine and HCs from the MRI Core of the Registry for Migraine (REFORM) study [19, 20]. Detailed descriptions of the migraine cohort are available in prior REFORM publications [20]. A previous report also presents fMRI findings following visual stimulation in participants with migraine and its subtypes, relative to HCs [10].

### Design and participants

This cross-sectional fMRI study enrolled adults with persistent PTH, migraine, or HCs. Participants with persistent PTH or migraine were recruited from a national referral hospital outpatient clinic, identified via medical records or specialist referrals. HCs were recruited through advertisements posted on a Danish research participation website (https://forsoegsperson.dk/).

Inclusion and exclusion criteria for each group are detailed in Supplementary Tables 1–3. Participants with persistent PTH met the International Classification of Headache Disorders, 3rd edition (ICHD-3), criteria for persistent headache attributed to mild traumatic brain injury. They also had to report at least four monthly headache days in the previous three months and had no history of more than one TBI. Exclusion criteria for the persistent PTH group included prior whiplash injury, any primary headache disorder aside from infrequent episodic tension-type headache, and comorbid neurological or severe somatic disease.

Participants with migraine met ICHD-3 criteria for migraine without aura, migraine with aura, or chronic migraine [2]. Inclusion also required at least four migraine days per month over the past three months. Key exclusions included any comorbid neurological or serious somatic disease.

HCs had no personal history of primary or secondary headache disorders, except infrequent episodic tension-type headache. Additional criteria required no first-degree relatives with primary headache disorders, no regular medication use, and no clinically significant medical conditions.

### Procedures

Before MRI acquisition, participants completed a physical and neurological examination. A semi-structured interview assessed characteristics specific to persistent PTH or migraine. We recorded the presence of headache, associated symptoms such as photophobia, phonophobia, and nausea, and the nature of the headache both before and during the scan. Additional variables included time since last headache, migraine, migraine-like headache, aura, and menstruation.

To minimize pharmacological effects on the BOLD signal, participants avoided analgesics, acute headache treatments, anti-inflammatory or antihistaminic drugs, and sedatives for 48 h before scanning. They were also instructed to abstain from caffeine-containing foods and beverages for 12 h prior to MRI.

### MRI procedures

All imaging was conducted using a 3.0 Tesla Siemens MAGNETOM Prisma scanner (Siemens Healthineers, Erlangen, Germany) with a 32-channel head coil. To minimize head motion, participants were instructed to remain still, and foam pads were placed around the temples.

The full imaging protocol included structural and functional sequences. For this study, three primary sequences were analyzed. First, task-based fMRI was used to capture BOLD responses to visual stimuli. Second, a gradient recalled echo (GRE) sequence was included to correct for magnetic field inhomogeneities. Third, a magnetization-prepared rapid acquisition gradient echo (MPRAGE) sequence supported structural co-registration.

The task-based fMRI parameters were: voxel size 3.0 × 3.0 × 3.0 mm, repetition time 2000 ms, echo time 230 ms, field of view 230 × 230 × 126 mm, and 35 interleaved slices. For GRE, the voxel size was 3.0 × 3.0 × 3.0 mm, repetition time 400 ms, echo times 4.92 ms (first echo) and 7.38 ms (second echo), with a field of view of 230 × 230 × 126 mm and 35 interleaved slices. MPRAGE parameters were: voxel size 1.0 × 1.0 × 1.0 mm, repetition time 2300 ms, echo time 2.0 ms, inversion time 900 ms, field of view 263 × 350 × 350 mm, and 208 interleaved slices. Additional sequences, not reported here, will be discussed in future publications. Total scan time was 55 min.

### Visual stimulation paradigm

**Fig. 1:**
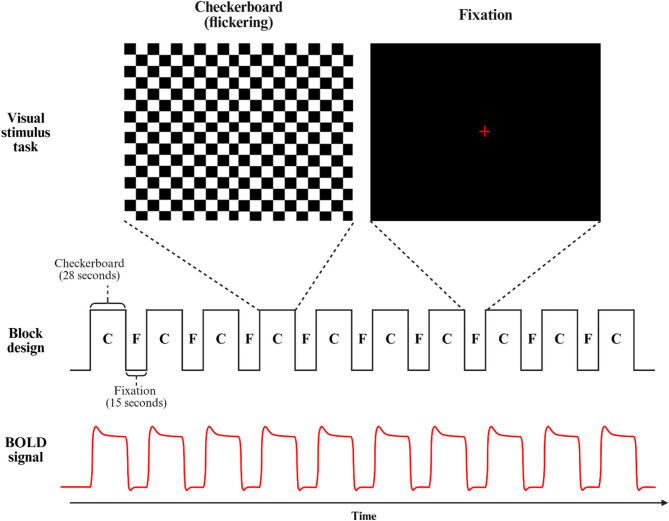
Block design of visual stimulation task with flickering checkerboard and fixation

We applied a block-design visual stimulation paradigm consisting of alternating periods of visual stimulation and fixation (Fig. 1). Visual stimulation blocks featured a flickering checkerboard pattern. Each stimulation block lasted 28 s, followed by a 15-second fixation block. The checkerboard reversed contrast every 0.11 s and alternated between wide and narrow formats every 0.35 s. The paradigm included 10 stimulation blocks and 11 fixation blocks, yielding a total duration of 445 s.

Participants were instructed to fixate on the center of the screen throughout the session. They were reminded of this instruction immediately before the paradigm began. No other cognitive or sensory tasks were presented during scanning. After the session, participants were asked whether they had maintained fixation throughout. Participants who failed to maintain gaze or showed inconsistent BOLD responses were excluded from further analysis.

### Preprocessing

MRI data were preprocessed using SPM12 (Wellcome Centre for Human Neuroimaging, London) implemented in MATLAB. The pipeline included field map correction using phase and magnitude images. Subsequent steps included realignment, slice timing correction, and co-registration to the MPRAGE structural volume. Data were normalized to the MNI152 template and smoothed with an 8 mm full-width at half maximum Gaussian kernel.

For first-level analysis, we modeled the contrast between rest and visual stimulation blocks. This contrast was convolved with the hemodynamic response function using a general linear model. Motion-related noise was controlled by including six motion regressors (three translational and three rotational). Volumes were flagged as motion outliers if they exceeded 0.9 mm displacement or deviated more than 5 standard deviations from the global signal. We identified outliers using the Artifacts Detection Tools (ART) in SPM12. Participants with 15% or more outlier volumes were excluded from further analysis.

### Participant classification: categories and subgroups

Participants with persistent PTH were divided into two subgroups based on photophobia at the time of scanning: those experiencing photophobia and those who were not. Some participants reported persistent and unremitting photophobia unrelated to headache. In these cases, photophobia was only recorded when its intensity increased alongside headache intensity, as previously defined [4, 5].

Participants with migraine were classified using a tiered system based on three dimensions. The categories were not mutually exclusive, allowing participants to be assigned to more than one. However, the subgroups within each category were mutually exclusive. First, participants were categorized by migraine frequency as either episodic or chronic. Second, they were classified by aura status. Participants who had ever experienced aura symptoms were placed in the migraine with aura subgroup, even if they also reported attacks without aura. Third, headache status at the time of scanning was recorded. Participants were classified as “ictal” if experiencing a migraine attack, “non-migraine headache” if symptoms did not meet migraine criteria, or “headache-free” if no headache was present. Migraine attacks were defined using ICHD-3 criteria [2], except for the 4–72-hour duration criterion, which was excluded to include attacks at early onset.

### Outcome and outcome measures

The primary outcome was the BOLD response to visual stimulation, quantified as the beta weight in task-based fMRI. Outcomes included whole-brain and region-of-interest (ROI) comparisons across participant groups.

**Fig. 2:**
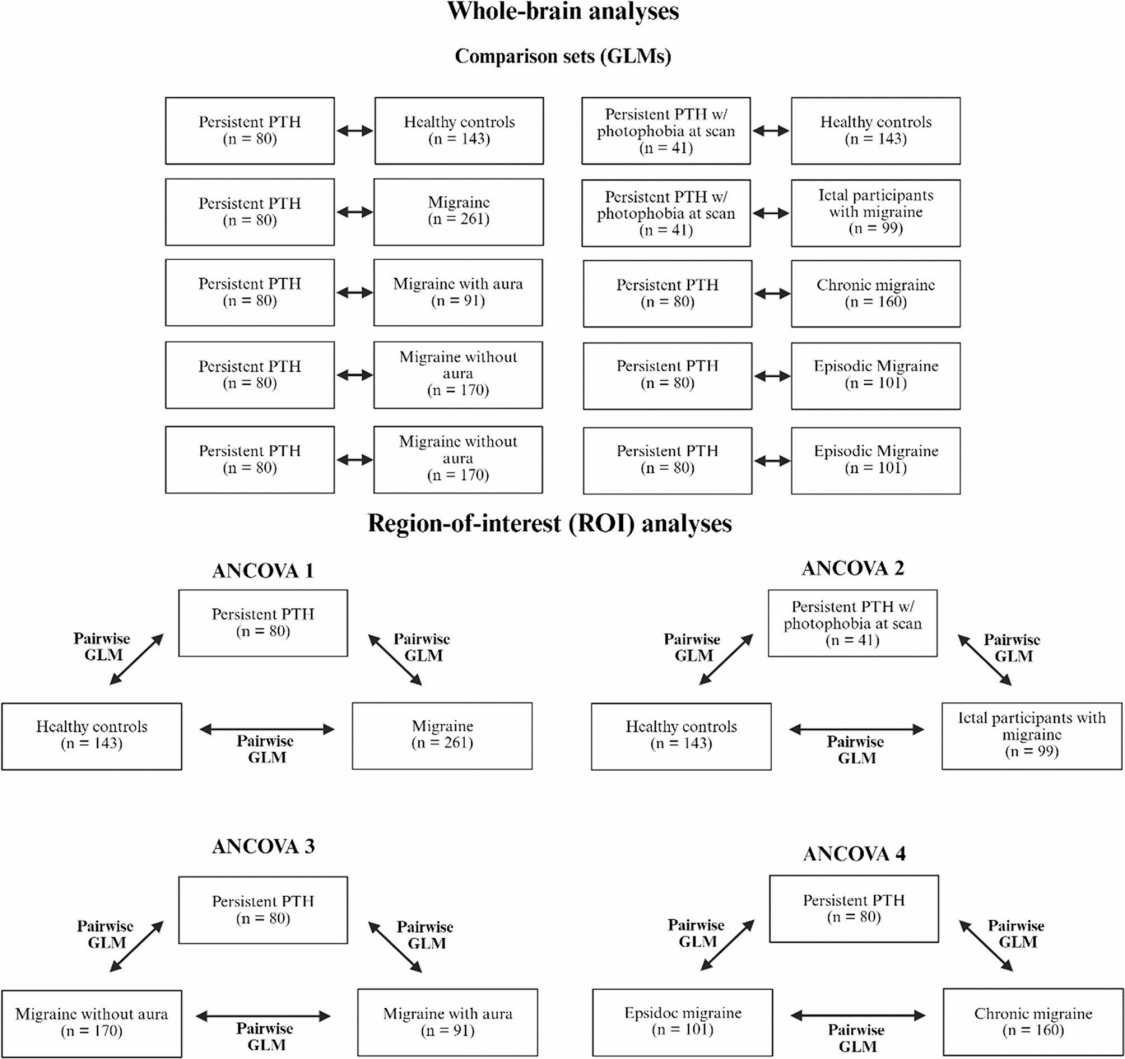
Overview of statistical comparisons for the whole brain and region-of-interest (ROI) analyses. For whole-brain analyses, groups were compared using GLMs as permutation testing thresholded at voxelwise *P *< 0.001, with clusterwise *P *< 0.05, familywise-error corrected for multiple comparisons. For region-of-interest (ROI) analyses, averaged BOLD responses within ROIs were initially compared in ANCOVAs. If an ANCOVA for a ROI was significant, pairwise GLMs were performed between the ANCOVA groups, to determine which group the difference derived from. For ROI ANCOVAs and pairwise GLMs, significance was accepted at *P *< 0.05. The ROIs included the bilateral cephalic region of the postcentral gyrus (see methods for coordinates), anterior cingulate cortex, insula, cuneus, lingual gyrus, thalamus, and hypothalamus (see methods for coordinates). Post-hoc, we also investigated ROI differences for the middle-cingulate cortex specifically in ANCOVA 2, pertaining to photophobia. Unless otherwise noted, the ROIs were derived from the Talairach Daemon of the WFU Pickatlas. All analyses were adjusted for age and sex. ANCOVA; analysis of covariance, GLM; general linear model, PTH; post-traumatic headache

We analyzed BOLD responses in the following comparisons (Fig. 2):

(I) Participants with persistent PTH, participants with migraine, and HCs;
(II) Participants with persistent PTH and photophobia during the scan, ictal participants with migraine, and HCs;
(III) Participants with persistent PTH, participants with migraine with aura, and those with migraine without aura;
(IV) Participants with persistent PTH, chronic migraine, and episodic migraine.

Moreover, a sensitivity analysis of comparison (II) was conducted, focusing on participants with persistent PTH who were photophobic during the scan, ictal participants with migraine who were also photophobic during the scan, and HCs.

**Fig. 3:**
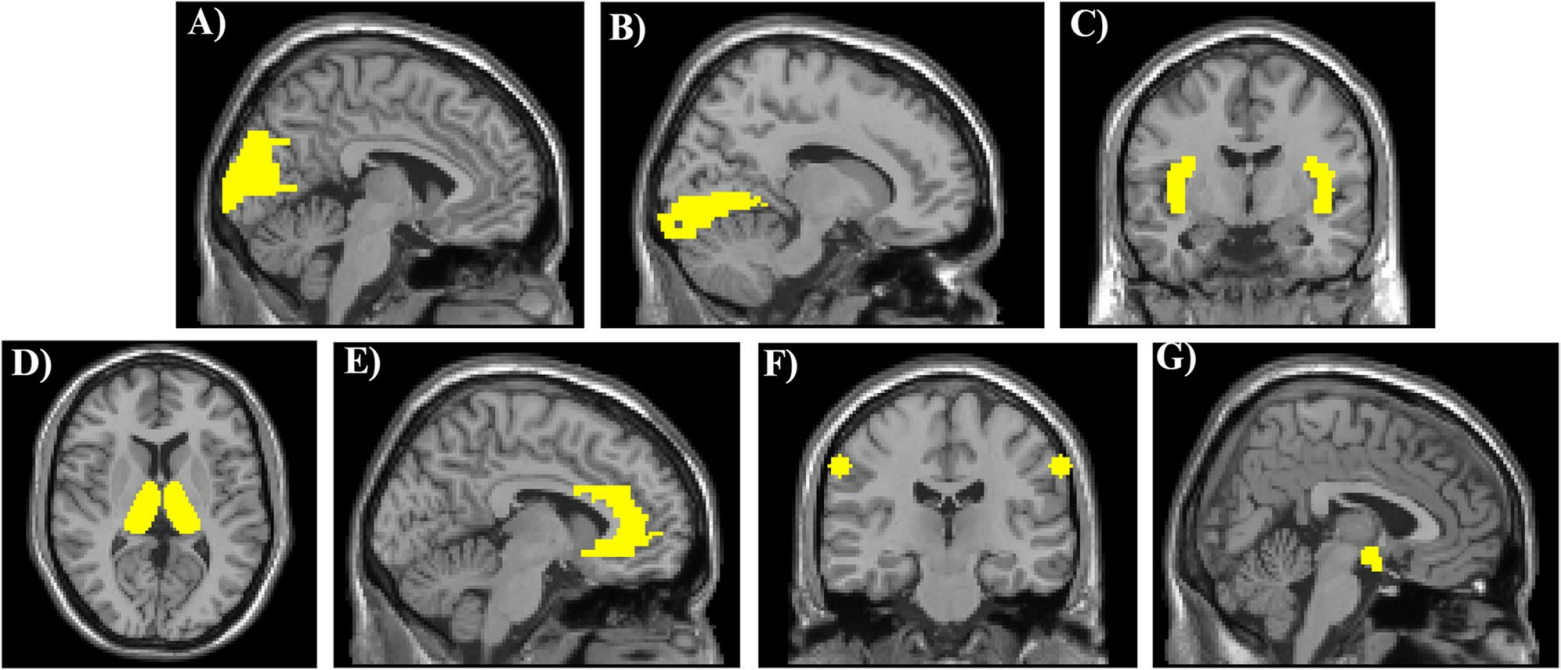
A priori regions of interest based on the Talairach Daemon (WFU pickatlas), which were used for secondary outcomes. From left to right: **A** cuneus, **B** lingual gyrus, **C** insula, **D** thalamus, **E** anterior cingulate cortex, **F **cephalic region of postcentral gyrus (coordinates from prior study), **G** hypothalamus (coordinates from prior study)

ROIs were selected based on the available literature implicating these regions in the neurobiological underpinnings of migraine and persistent PTH [7, 18, 21, 22]. Bilateral ROIs were defined using the Wake Forest University (WFU) PickAtlas and included the cuneus, lingual gyrus, anterior cingulate cortex, postcentral gyrus, hypothalamus, insula, and thalamus (Fig. 3).

For the postcentral gyrus, we created a custom ROI in the bilateral cephalic region of the sensory homunculus. This was defined using a 10 mm radius centered at MNI coordinates: x = −62, y = −14, z = 40 (right), and x = 62, y = −14, z = 40 (left) [23]. The bilateral hypothalamic ROI was modeled using a 6 mm sphere centered at ± 6, −6, 10, based on peak coordinates from a prior migraine study [24].

As a post-hoc analysis, we also examined BOLD differences related to photophobia within the midcingulate cortex. This ROI was derived from the middle cingulate WFU PickAtlas region and labeled separately from the anterior cingulate cortex to ensure anatomical distinction.

### Statistical analysis

No formal sample size calculations were performed. However, we aimed to include more than 70 participants with persistent PTH, a sample size exceeding that of any previously published fMRI investigations in this population. This enrollment target was intended to enhance the accuracy and reproducibility of the findings. Clinical data were analyzed using *R* (version 4.1.0). Continuous variables were summarized as mean ± standard deviation if normally distributed, or as median with interquartile range if not. Normality was assessed using Shapiro-Wilk’s test. Group comparisons for normally distributed variables were conducted using unpaired t-tests. For non-normally distributed variables, we used Mann-Whitney U tests. Age distributions were compared using Kolmogorov-Smirnov’s test. Categorical variables were presented as counts with percentages and compared using the χ²-test.

Imaging data were processed and analyzed in SPM12 and MATLAB. We used the WFU PickAtlas and Statistical nonParametric Mapping (SnPM) toolboxes. We applied a data-driven approach and did not perform a formal sample size calculation. Whole-brain analyses were conducted using general linear model permutation tests with 5000 iterations. We applied a voxel-wise threshold of *P* ≤ 0.001 and corrected for multiple comparisons using familywise error (FWE) at *P* ≤ 0.05. All models were adjusted for age and sex.

ROI analyses were conducted using analyses of covariance (ANCOVAs) adjusted for age and sex. When ANCOVA results were significant, we followed up with pairwise general linear models to assess group differences. For significant ROIs, beta values were extracted post hoc and tested for associations with clinical variables using Pearson’s or Spearman’s correlation tests, as appropriate. For all ANCOVAs, GLMs, and correlation tests, we considered results significant at *P* ≤ 0.05.

## Results

Between November 2020 and October 2023, we enrolled and scanned 105 participants with persistent PTH, 306 with migraine, and 160 HCs. After exclusions due to head motion or incidental findings, 80 participants with persistent PTH, 261 with migraine, and 143 HCs were included in the final analysis. Demographic and clinical data are presented in Table 1.

**Table 1:** Demographic and clinical characteristics of the study populations

Characteristics	Persistent PTH	Healthy controls	Migraine	*P*-values (Persistent PTH vs. Healthy controls)	*P*-values (Persistent PTH vs. Migraine)
No.	80	143	261	-	-
Male: female, n	19:61	20:123	29:232	0.656	0.005*
Age, mean (SD)	41.4 (11.0)	41.6 (11.7)	41.1 (12.3)	0.777	0.812
Right-handed, n (%)	72 (90.0)	123 (86.0)	244 (93.5)	0.540	0.491
Migraine with aura, n (%)	NA	NA	89 (34.5)	NA	NA
Chronic migraine, n (%)	NA	NA	157 (60.2)	NA	NA
Monthly migraine-like days and migraine days, mean (SD)	13.9 (11.9)	NA	13.2 (6.9)	NA	0.776
Monthly headache days, mean (SD)	25.7 (8.2)	NA	18.9 (8.3)	NA	< 0.001*
Days with acute medication, mean (SD)	9.4 (8.9)	NA	11.7 (6.4)	NA	< 0.001*
Using preventive treatment, n (%)	33 (41.3)	NA	138 (52.9)	NA	0.069
Medication-overuse headache, n (%)	11 (13.8)	NA	91 (34.5)	NA	< 0.001*
Disease duration, years, mean (SD)	8.5 (5.5)	NA	22.0 (12.0)	NA	< 0.001*
Comorbid depression, n (%)	28 (35.0)	NA	36 (13.8)	NA	< 0.001*
Comorbid anxiety, n (%)	19 (23.8)	NA	30 (11.5)	NA	0.006*
*Phase during scan*					
Ictal, n (%)†	6 (7.5)	NA	73 (28.0)	NA	< 0.001*
Non-migrainous headache, n (%)††	68 (85.0)	NA	107 (41.0)	NA	< 0.001*
Headache free, n (%)	6 (7.5)	NA	81 (31.0)	NA	< 0.001*

*Significant at *P* < 0.05

†Headache fulfilling criteria for an attack of migraine without aura, according to the ICHD-3

††Headache not fulfilling criteria for an attack of migraine without aura, according to the ICHD-3

Participants with persistent PTH had a mean age of 41.4 ± 11.0 years, and 61 (77.6%) were female. They reported an average of 25.7 ± 8.2 headache days per month. A migraine-like phenotype was present in 77 (96.3%) participants. Eleven (13.8%) met criteria for medication-overuse headache, and 33 (41.3%) used preventive headache medication. During scanning, 74 (92.5%) reported ongoing headache, and 41 (51.3%) reported photophobia.

Participants with migraine had a mean age of 41.1 ± 12.3 years, with 232 (88.9%) female. They reported 18.9 ± 8.3 headache days per month. Chronic migraine was present in 157 (60.2%) participants, and 89 (34.5%) had migraine with aura. During the scanning, 73 (28.0%) participants were ictal, 107 (41.0%) had a non-migraine headache, and 81 (31.07%) were headache-free. When including data from follow-up fMRI sessions, the final sample comprised 99 participants with ictal scans, among whom 84 (84.8%) reported experiencing photophobia at the time of imaging.

The HC group had a mean age of 41.6 ± 11.7 years, with 123 (86.0%) female. No significant age differences were observed between groups (all *P* > 0.05). However, the persistent PTH group included a higher proportion of males than the migraine (*P* < 0.01) but not the HC group (*P* = 0.07). Sex distribution was similar between the migraine and HC groups (*P* = 0.72).

### Whole-brain analysis

No significant differences in BOLD responses were found between the whole groups of participants with persistent PTH, participants with migraine, and HCs. Likewise, no differences were observed between participants with persistent PTH and migraine subgroups, including migraine with aura, migraine without aura, chronic migraine, or episodic migraine.

### Photophobia in participants with persistent PTH

**Fig. 4:**
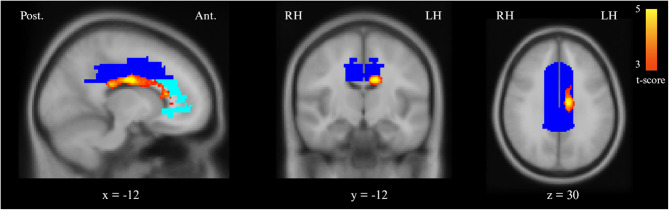
Regions with greater BOLD response in photophobic participants with persistent PTH compared with HCs.Images in radiological convention. Greater BOLD response in cluster in the left midcingulate cortex, anterior cingulate cortex, and corpus callosum (*P*_FWE_*= *0.010), compared with HCs in whole-brain analysis (warm colors). Cluster extend = 728 mm^3^, peak MNI coordinates: X=−12, Y=−10, Z=28. Voxelwise threshold: *P *< 0.001, clusterwise threshold: *P* < 0.05, familywise error corrected. Displayed overlapping Talairach Daemon labels for the middle cingulate cortex (blue) and anterior cingulate cortex (light blue). LH; left hemisphere, RH; right hemisphere

Participants with persistent PTH who experienced photophobia during scanning showed greater BOLD response in a cluster spanning the left midcingulate cortex, anterior cingulate cortex, and adjacent corpus callosum. This activation differed significantly from HCs (*P*_*FWE*_ = 0.010; Table 2, Fig. 4). The cluster followed the curvature of the anterior and midcingulate cortex. No significant differences in BOLD signal were identified when compared this group to ictal participants with migraine. A separate sensitivity analysis also found no differences to ictal participants with migraine who experienced photophobia during the fMRI scan.

**Table 2:** Whole-brain differences between photophobic participants with persistent PTH and HC. Differences in BOLD response in cluster-based whole-brain analysis between participants with persistent PTH who reported photophobia during the scan, compared to hcs. Permutation analyses adjusted for age and sex

Comparison	Cortical region	Cluster *P*-value (FWE)	Extent of area (k)	MNI coordinates (peak voxel)		
				X	Y	Z
Photophobic participants with persistent PTH vs. HC	Left mid cingulate cortex, anterior cingulate cortex, and corpus callosum	0.010	728	−12	−10	28

*FWE* Familywise-error, *HC* Healthy controls, *MNI* Montreal Institute of Neurology, *PTH* Post-traumatic headache

### Region of interest analysis

ROI analyses revealed a significant group effect in the cuneus (*P* = 0.011) (Table 3). Post hoc comparisons showed that participants with migraine had a greater cuneal BOLD response than HCs (*P* = 0.006). No significant differences were observed between participants with persistent PTH and either the migraine group or HCs (both *P* > 0.05). Furthermore, participants with persistent PTH did not differ from any migraine subgroups in BOLD responses (Supplementary Tables 5–6).

**Table 3:** Region of interest (ROI) differences between participants with persistent PTH, migraine, and HC. Regional differences in BOLD response analyzed with analysis of covariance (ANCOVA) adjusted for age and sex

Comparison	Cerebral region	F-value	*P*-value
Participants with persistent PTH vs. migraine vs. HC	Bilateral postcentral gyrus (cephalic region)	0.967	0.381
	Bilateral anterior cingulate cortex	0.283	0.754
	Bilateral insula	1.610	0.201
	Bilateral cuneus	4.603	0.011*
	Bilateral lingual gyrus	0.375	0.688
	Bilateral thalamus	0.232	0.793
	Bilateral hypothalamus	1.161	0.314

*HC* Healthy controls, *PTH* Post-traumatic headache

*Significant at *P* < 0.05

### Photophobia in participants with persistent PTH: region of interest analysis

ROI analysis revealed significant group effects in the insula, anterior cingulate cortex, midcingulate cortex (post-hoc), and thalamus (Table 4). Photophobic participants with persistent PTH showed greater BOLD responses than HCs in the insula (*P* = 0.033), anterior cingulate cortex (*P* = 0.029), and midcingulate cortex (*P* = 0.014). Likewise, ictal participants with migraine had greater BOLD responses than HCs in the insula (*P* = 0.009), anterior cingulate cortex (*P* = 0.017), midcingulate cortex (*P* = 0.018), and thalamus (*P* = 0.020). No significant differences were observed between photophobic participants with persistent PTH and ictal participants with migraine. In additional sensitivity analyses, there were likewise no differences between photophobic participants with persistent PTH and ictal participants with migraine who were photophobic during the scan. When compared with healthy controls, the ictal participants with migraine who were photophobic exhibited greater BOLD responses within the same ROIs as the ictal (bilateral anterior cingulate cortex, midcingulate cortex, insula, and thalamus), as well as within the cephalic region of the postcentral gyrus (*P* = 0.048; Supplementary Tables 7–8).

**Table 4:** Region of interest (ROI) differences between photophobic persistent PTH, ictal migraine, and HC. Regional differences in BOLD response between participants with persistent PTH who were photophobic during the scan, participants with migraine who were ictal during the scans, and hcs. Analyzed with analysis of covariance (ANCOVA) adjusted for age and sex

Comparison	Cerebral region	F-value	*P*-value
Participants with persistent PTH who were photophobic during the scan vs. ictal participants with migraine vs. HC	Bilateral postcentral gyrus (cephalic region)	1.658	0.058
	Bilateral anterior cingulate cortex	3.109	0.011*
	Bilateral mid-cingulate cortex (post-hoc analysis)	4.743	0.009*
	Bilateral insula	3.916	0.008*
	Bilateral cuneus	1.619	0.187
	Bilateral lingual gyrus	0.120	0.747
	Bilateral thalamus	2.880	0.028*
	Bilateral hypothalamus	1.249	0.332

*HC* Healthy controls, *PTH* Post-traumatic headache

*Significant at *P* < 0.05

## Discussion

This fMRI study aimed to determine whether persistent PTH shares functional neural mechanisms with migraine, particularly when photophobia is present. Our findings support this hypothesis while also delineating points of divergence between the two headache disorders. The whole-brain analyses revealed no differences in BOLD responses among the full persistent PTH, migraine, and HCs groups. However, photophobic participants with persistent PTH exhibited greater BOLD responses in anterior cingulate, midcingulate, and insular cortices, compared with HCs. These same regions also showed increased BOLD responses in ictal participants with migraine, when compared to HCs [10]. Their engagement in both headache disorders suggests the existence of a shared neural circuit underlying photophobia.

### Shared neural correlates of photophobia in persistent PTH and migraine

Our results highlight the anterior cingulate cortex and insula as key sites of neural convergence during photophobia in both persistent PTH and migraine. These regions integrate nociceptive, emotional, and interoceptive input and are fundamental to pain perception [11–13, 25, 26]. Both the anterior cingulate and insular cortices are consistently activated in response to noxious stimuli and contribute to the emotional salience of pain [12, 27–29]. Their co-activation during photophobia suggests that, under certain conditions, light is reclassified by the brain as an aversive sensory input [30]. This re-interpretation of light as noxious might explain the heightened discomfort patients report during light exposure in both persistent PTH and migraine [30]. Importantly, both whole-brain and ROI analyses implicated the midcingulate cortex, supporting the involvement of a broader pain processing network [12, 29, 31]. Moreover, the clinical relevance of these regions is well established. For example, partial cingulotomy targeting the anterior cingulate has shown benefit in refractory cancer pain, underscoring its role in the modulation of affective pain components [32].

The activation of cingulate and insular regions during photophobia likely reflects the transformation of visual stimuli into pain-exacerbating signals. This offers a neural explanation for why light can worsen headache intensity in people with persistent PTH and migraine [30]. Ample preclinical evidence supports this concept. Animal studies show that trigeminal and visual afferents converge within multimodal neurons of the posterior thalamus [33, 34], which then project to pain-related cortical areas [30, 34]. This circuitry provides a mechanistic bridge between light exposure and headache amplification. In the present study, ictal participants with migraine also exhibited thalamic hyperexcitability consistent with this preclinical framework. Although the same trend was observed in participants with persistent PTH, it did not reach significance (*P* = 0.06). This might reflect sample size limitations or signal dilution due to averaging across thalamic subnuclei.

In combination with established preclinical models of photophobia [30], the present findings suggest a clinically meaningful overlap in neural mechanisms underlying hypersensitivity to light in both migraine and persistent PTH. Despite their distinct etiologies, both headache disorders might share a convergent pathways involving thalamocortical processing of trigemino-visual input. This supports the hypothesis that light-induced headache exacerbation in these patient populations arises, at least in part, from a shared dysfunction in multimodal sensory integration.

The clinical implications of this shared circuitry are substantial. If thalamic and cortical structures mediate photophobia via converging afferent input, then therapeutic interventions targeting trigeminal activation might alleviate photophobia in both migraine and persistent PTH. This possibility is supported by clinical observations showing concurrent improvement in migraine severity and photophobia after treatment with acute medications [35]. Whether similar therapeutic responses occur in persistent PTH remains to be determined.

An alternative hypothesis is that thalamic neurons could continue to activate photophobia-related cortical regions even after peripheral trigeminal input has been reduced. This could explain why prodromal photophobia often persists beyond headache resolution in migraine [36], and might also be relevant in persistent PTH. To clarify the temporal dynamics and mechanistic basis, future studies should use functional neuroimaging and preclinical models designed to assess visual system responsivity after therapeutic intervention. Together, our findings offer a coherent neurobiological model for photophobia. They link a subjective symptom to objective patterns of brain activation. The recruitment of shared neural networks in both headache disorders supports the existence of a shared photophobia circuit.

### Visual cortex hyperexcitability: a distinctive feature of migraine

Our study identifies visual cortex hyperexcitability as a pathophysiological feature specific to migraine and absent in persistent PTH, despite phenotypic similarities between the two disorders. Participants with migraine showed increased BOLD activation in the cuneus during visual stimulation, regardless of attack status. In contrast, participants with persistent PTH did not differ from HCs in visual cortex responsiveness, even when photophobic.

One possible explanation is the acquired nature of persistent PTH compared to the genetic origins of migraine. Familial aggregation studies and genome-wide association data support a heritable predisposition to cortical hyperexcitability in migraine [37, 38]. In contrast, persistent PTH arises from traumatic injury and might not confer the same neurophysiological architecture.

Taken together, our findings suggest that visual cortex hyperresponsivity reflects a stable, disease-specific characteristic of migraine. Its absence in persistent PTH provides mechanistic support for distinct pathophysiological trajectories, despite shared clinical features.

### Strengths and limitations

A principal strength of this study is its large, well-characterized sample, comprising participants with persistent PTH, participants with migraine, and HCs. All analyses were adjusted for age and sex, reducing confounding from demographic variables. The lack of an upper age limit should likewise increase the generalizability of our results. We applied validated whole-brain analytical methods with cluster correction to control false-positive rates, consistent with best-practice recommendations [39, 40]. Another strength lies in the real-world diversity of our migraine sample, which provided imaging data on multiple migraine subtypes and attack phases.

Nonetheless, several limitations should be noted. First, the cross-sectional design limits causal inference; we cannot determine whether the identified increases in BOLD responses precede or follow the onset of photophobia. Second, symptom ratings—including photophobia—were based on self-report, which might introduce bias. Photophobia itself can encompass various phenomena, including light-induced exacerbation of headache, abnormal light sensitivity, ocular discomfort, or more generalized aversion to light [30]. Although the present study did not differentiate between these subtypes, future studies should aim to clarify the shared and distinct mechanisms underlying their expression. Third, although sex-matching was not achieved across all comparisons, our analyses adjusted for sex and age, making confounding unlikely to account for the observed group-level differences. Additional factors—including psychiatric comorbidities, headache frequency, and medication-overuse headache status—differed across groups and were not included as covariates. While these factors could theoretically influence neural responses, prior research has not demonstrated a consistent effect on neural activity during simple visual stimulation [41, 42], and our group comparisons were similar to those reported in all earlier studies that also did not adjust for these variables [15, 16, 18, 43, 44]. Fourth, the effect of ongoing headache or migraine-like headache in participants with persistent PTH was not specifically analyzed. This reflects the fact that the overwhelming majority of these participants (92.5%) reported headache at the time of scanning, with only a small minority (7.5%) experiencing migraine-like headache. As such, the available data either lacked sufficient variability or statistical power to support subgroup analysis. Future studies with larger or more heterogeneous samples may be better positioned to examine this factor in greater detail. Fifth, part of the activation cluster observed in photophobic participants with persistent PTH extended into adjacent white matter regions. This is likely attributable to spatial smoothing across tissue boundaries, a known effect in fMRI preprocessing [45]. Importantly, ROI analyses consistently confirmed involvement of the anterior and midcingulate cortices, supporting the validity of our primary findings despite partial white matter overlap. Lastly, while the sample size is large relative to prior fMRI studies in persistent PTH, it might still lack power to detect subtle effects. False negatives cannot be entirely excluded. However, task-based fMRI using robust sensory paradigms—such as visual stimulation—generally yields more reproducible activity patterns than resting-state functional connectivity [46, 47], thereby increasing confidence in the observed effects.

## Conclusions

This study provides compelling evidence for shared neural correlates of photophobia in persistent PTH and migraine, particularly within cingulate and insular cortices involved in pain and salience processing. At the same time, we identified visual cortex hyperexcitability as a distinguishing feature of migraine, underscoring divergent pathophysiological mechanisms despite overlapping clinical features. These findings advance our understanding of photophobia by linking it to both specific brain activation patterns.

## Supplementary Information

Supplementary Material 1.

## Data Availability

The corresponding author will provide relevant data upon reasonable request from a qualified investigator, for the purpose of academic scrutiny, reproducibility, and further scientific investigation.

## Abbreviations

ART: Artifacts Detection Tools
BOLD: Blood Oxygen Level-Dependent
fMRI: Functional Magnetic Resonance Imaging
FWE: Familywise Error
GRE: Gradient Recalled Echo
HCs: Healthy Controls
ICHD-3: International Classification of Headache Disorders, 3rd edition
MNI: Montreal Neurological Institute
MPRAGE: Magnetization-Prepared Rapid Acquisition Gradient Echo
MRI: Magnetic Resonance Imaging
mTBI: Mild Traumatic Brain InjuryPTH: Post-Traumatic Headache
REFORM: Registry for Migraine
ROI: Region of Interest
SD: Standard Deviation
SnPM: Statistical nonParametric Mapping
SPM: Statistical Parametric Mapping
TBI: Traumatic Brain Injury
WFU: Wake Forest University
