# Supplementary material for: Shared neural signatures of photophobia in migraine and post-traumatic headache: a task-based fMRI study
Source: J Headache Pain. 2025 Jul 3;26(1):154. doi: 10.1186/s10194-025-02088-y (PMC12231636; doi:10.1186/s10194-025-02088-y)
Supplement: Supplementary file 1 — Supplementary Material 1. [file 10194_2025_2088_MOESM1_ESM.docx]

**Supplementary Materials**

**Supplementary table 1. Eligibility criteria for participants with persistent post-traumatic headache.**

| **Inclusion criteria for participants with persistent post-traumatic headache** | **Exclusion criteria for participants with persistent post-traumatic headache** |
| --- | --- |
| - ≥ 18 years of age upon entry into screening - History of persistent headache attributed to mild traumatic injury to the head for ≥ 12 months according to the International Classification of Headache Disorders version 3^rd^ edition (ICHD-3) criteria (code 5.2.2). - ≥ 4 monthly headache days on average for 3 months prior to study screening - Provision of informed consent prior to initiation of any study-specific activities/procedures | - Suffered > 1 mild traumatic injury to the head - History of moderate or severe traumatic injury to the head. - History of whiplash injury - or any moderate to severe traumatic injury to the head. - History of craniotomy - History or evidence of any other clinically significant disorder, condition or disease (with the exception of those outlined above), that might pose a risk to subject safety or interfere with study evaluation. - Inability to differentiate migraine headache from other headaches - Risk of self-harm or harm to others as evidenced by past suicidal behavior - Female subjects of childbearing potential with a positive pregnancy test during any study visit - Evidence of current pregnancy or breastfeeding - Concomitant preventive medication apart from anti-CGRP antibodies was allowed, but only if dosage was stable for 2 months prior to the baseline period. |

**Supplementary table 2. Eligibility criteria for participants with migraine.** Modified from Christensen et al. *[19]*.

| **Inclusion criteria for participants with migraine** | **Exclusion criteria for participants with migraine** |
| --- | --- |
| - ≥ 18 years of age upon entry into screening - History of migraine with or without aura for ≥ 12 months according to the International Classification of Headache Disorders version 3^rd^ edition (ICHD-3) criteria (code 1.2 or 1.1) for at least one year prior to study entry - ≥ 4 monthly headache days that meet criteria as migraine days on average for 3 months prior to study screening - Provision of informed consent prior to initiation of any study-specific activities/procedures - Scheduled preventive treatment with erenumab | - > 50 years of age at migraine onset - History of persistent post-traumatic headache, hemiplegic migraine, or cluster headache (ICHD-3 code 5.2, 1.2.3, and 3.1) - Inability to differentiate migraine headache from other headaches - Risk of self-harm or harm to others as evidenced by past suicidal behavior - History or evidence of any other clinically significant disorder, condition or disease (with the exception of those outlined above), that might pose a risk to subject safety or interfere with study evaluation. - Previous treatment with erenumab - Treatment with another anti-CGRP monoclonal antibody for 3 months prior to first study visit - Concomitant preventive medication apart from anti-CGRP antibodies was allowed, but only if dosage was stable for 2 months prior to the baseline period. |

**Supplementary table 3. Eligibility criteria for healthy controls.** Modified from Christensen et al. *[19]*.

| **Inclusion criteria for healthy controls** | **Exclusion criteria for healthy controls** |
| --- | --- |
| - ≥ 18 years of age at the screening visit | - History of primary headache disorders (except infrequent episodic tension-type headache) - History of secondary headache disorders - Any first-degree relative with a history of any primary headache disorders (except ≤ 5 monthly days with tension-type headache) - Headache within 24 hours of any study-related task or procedure - Past suicidal behavior or risk of self-harm - Daily use of medications except oral contraceptives - History or evidence any other clinically significant disorder, condition, or disease that, in the opinion of the site investigator, would pose a risk to participant safety or interfere with study evaluation, procedures or completion - History or evidence of any psychiatric disorder - Female study participants of childbearing potential with a positive pregnancy test during the study visit - Pregnancy or breastfeeding, female study participants unwilling to use one acceptable method of effective contraception (oral contraceptives, intrauterine device, intrauterine hormonal-releasing system, bilateral tubal ligation/occlusion, vasectomized partner, sexual abstinence, male or female condom, cap, diaphragm, or sponge with spermicide) - Contraindications to MRI - Unlikely to comply with and complete all protocol-required procedures to the best of the study participant and the study investigator’s knowledge. |

**Supplementary table 4. Headache characteristics during scan in persistent PTH.**

| Characteristics and associated symptoms | Headache |
| --- | --- |
| No. | 80 |
| No. with headache | 74 (92.5) |
| No. with constant headache | 43 (53.8) |
| Hours from onset until scan, median (IQR)^b^ | 5 (3-8.3) |
| Intensity in NRS, median (IQR) | 4 (3-5) |
| Mild, n (%) | 23 (28.8) |
| Moderate, n (%) | 42 (52.5) |
| Severe, n (%) | 9 (11.3) |
| Unilateral, n (%) | 18 (22.5) |
| Pulsating, n (%) | 13 (16.3) |
| Aggravation by physical activity, n (%) | 25 (31.3) |
| Nausea and/or vomiting, n (%) | 13 (16.3) |
| Photophobia, n (%) | 41 (51.3) |
| Phonophobia, n (%) | 34 (42.5) |

**IQR=interquartile range.**

**^b^Excluding participants with constant headache**

**Supplementary table 5. Region of interest (ROI) differences between participants with persistent PTH, migraine without aura, and migraine with aura.** Regional differences in BOLD response analyzed with analysis of covariance (ANCOVA) adjusted for age and sex.

| Comparison | Cerebral region | *F-*value | *P-*value |
| --- | --- | --- | --- |
| Participants with persistent PTH vs migraine without aura vs migraine with aura | Bilateral postcentral gyrus (cephalic region) | 0.113 | 0.893 |
|  | Bilateral anterior cingulate cortex | 0.012 | 0.989 |
|  | Bilateral insula | 0.578 | 0.562 |
|  | Bilateral cuneus | 2.047 | 0.131 |
|  | Bilateral lingual gyrus | 0.396 | 0.674 |
|  | Bilateral thalamus | 0.180 | 0.835 |
|  | Bilateral hypothalamus | 1.111 | 0.330 |

**Supplementary table 6. Region of interest (ROI) differences between participants with persistent PTH, chronic migraine, and episodic migraine.** Regional differences in BOLD response analyzed with analysis of covariance (ANCOVA) adjusted for age and sex.

| Comparison | Cerebral region | *F-*value | *P-*value |
| --- | --- | --- | --- |
| Participants with persistent PTH vs chronic migraine vs episodic migraine | Bilateral postcentral gyrus (cephalic region) | 0.224 | 0.800 |
|  | Bilateral anterior cingulate cortex | 1.346 | 0.262 |
|  | Bilateral insula | 0.638 | 0.529 |
|  | Bilateral cuneus | 2.162 | 0.117 |
|  | Bilateral lingual gyrus | 0.623 | 0.534 |
|  | Bilateral thalamus | 0.008 | 0.992 |
|  | Bilateral hypothalamus | 1.443 | 0.238 |

***Significant at *P* < 0.05.**

**Supplementary table 7.** ANCOVA comparisons for significant variance between photophobic participants with persistent PTH, and photophobic ictal participants with migraine, as well as HCs. The presence of photophobia was registered at the time of scan.

| Comparison | Cerebral region | *F-*value | *P-*value |
| --- | --- | --- | --- |
| Photophobic participants with persistent PTH vs photophobic ictal participants with migraine vs HC | Bilateral postcentral gyrus (cephalic region) | 1.658 | **0.048*** |
|  | Bilateral anterior cingulate cortex | 3.109 | **0.013*** |
|  | Bilateral midcingulate cortex  (post-hoc analysis) | 4.743 | **0.015*** |
|  | Bilateral insula | 3.916 | **0.017*** |
|  | Bilateral cuneus | 1.619 | 0.105 |
|  | Bilateral lingual gyrus | 0.120 | 0.813 |
|  | Bilateral thalamus | 2.880 | **0.026*** |
|  | Bilateral hypothalamus | 1.249 | 0.365 |

HC, Healthy controls; PTH, post-traumatic headache.

***Significant at *P* < 0.05.**

**Supplementary table 8.** Pairwise comparisons focusing on regions significant in ANCOVAs (see Supplementary Table 7) between photophobic participants with persistent PTH, photophobic ictal participants with migraine, as well as HCs. The presence of photophobia was registered at the time of scan.

| Cerebral region | Photophobic PTH vs Photophobic Ictal Migraine  (*P*-value) | Photophobic PTH vs HCs  (*P*-value) | Photophobic Ictal Migraine vs HCs  (*P*-value) |
| --- | --- | --- | --- |
| Bilateral postcentral gyrus (cephalic region) | 0.735 | 0.058 | **0.047*** |
| Bilateral anterior cingulate cortex | 0.893 | **0.029*** | **0.021*** |
| Bilateral midcingulate cortex  (post-hoc analysis) | 0.639 | **0.014*** | **0.034*** |
| Bilateral insula | 0.967 | **0.033*** | **0.020*** |
| Bilateral thalamus | 0.943 | 0.062 | **0.018*** |

HC, Healthy controls; PTH, post-traumatic headache.

***Significant at *P* < 0.05.**
